# Grasslands, Invertebrates, and Precipitation: A Review of the Effects of Climate Change

**DOI:** 10.3389/fpls.2016.01196

**Published:** 2016-08-05

**Authors:** Kirk L. Barnett, Sarah L. Facey

**Affiliations:** Hawkesbury Institute for the Environment, Western Sydney University, PenrithNSW, Australia

**Keywords:** climate change, drought, insects, invertebrate communities, irrigation, rain-exclusion shelters, rainfall

## Abstract

Invertebrates are the main components of faunal diversity in grasslands, playing substantial roles in ecosystem processes including nutrient cycling and pollination. Grassland invertebrate communities are heavily dependent on the plant diversity and production within a given system. Climate change models predict alterations in precipitation patterns, both in terms of the amount of total inputs and the frequency, seasonality and intensity with which these inputs occur, which will impact grassland productivity. Given the ecological, economic and biodiversity value of grasslands, and their importance globally as areas of carbon storage and agricultural development, it is in our interest to understand how predicted alterations in precipitation patterns will affect grasslands and the invertebrate communities they contain. Here, we review the findings from manipulative and observational studies which have examined invertebrate responses to altered rainfall, with a particular focus on large-scale field experiments employing precipitation manipulations. Given the tight associations between invertebrate communities and their underlying plant communities, invertebrate responses to altered precipitation generally mirror those of the plants in the system. However, there is evidence that species responses to future precipitation changes will be idiosyncratic and context dependent across trophic levels, challenging our ability to make reliable predictions about how grassland communities will respond to future climatic changes, without further investigation. Thus, moving forward, we recommend increased consideration of invertebrate communities in current and future rainfall manipulation platforms, as well as the adoption of new technologies to aid such studies.

## Introduction

Grasses cover more of the earth’s surface than any other vegetation type (Tscharntke and Greiler, 1995; Wang and Fang, 2009) and are often of high economic, ecological and biodiversity value, providing forage for livestock and high levels of carbon storage (Lee et al., 2014; Lenhart et al., 2015). Many grasslands exist in seasonal states of water limitation, and are highly responsive to changes in water availability in terms of biomass and composition (Knapp et al., 2002; Fry et al., 2014; Lenhart et al., 2015). Climate models predict changes in precipitation patterns, in terms of the total amount and the frequency and intensity of rainfall events (IPCC, 2013), therefore leading to alterations in grassland plant composition and primary production.

Invertebrates are the most diverse and abundant constituent of terrestrial ecosystem fauna (Stork et al., 2015). Often overlooked, these organisms contribute to structuring grassland communities, through activities such as pollination and nutrient cycling (Whiles and Charlton, 2006) and contribute to shaping grasslands through top-down processes. For instance, herbivores can modify plant species richness by altering competitive dynamics between plant species (Olff and Ritchie, 1998). Likewise, plant community composition plays a bottom-up role in structuring arthropod communities (Perner et al., 2005; Hertzog et al., 2016), as do abiotic factors like temperature and water availability (e.g., Bale et al., 2002). Thus, both grassland plant and invertebrate communities can be directly impacted by alterations in climate. In addition, precipitation changes can have indirect impacts on both plants and invertebrates as the interactions occurring between the two communities are also climate-sensitive; the effect of herbivory on plant diversity varies across precipitation gradients, for instance (Olff and Ritchie, 1998).

It is in our interest to understand how climate change-driven alterations in precipitation will affect valuable grassland systems and the invertebrate communities they both support and rely on. Over the past 20 years, multiple experiments and observational studies have addressed the responses of grasslands to changes in precipitation, with a subset of these also examining invertebrate responses across a range of precipitation scenarios and spatial scales (summarized in **Table 1**). To our knowledge, there has been no synthesis of the relevant literature examining insect responses to precipitation changes, making a review of these studies timely. In this mini-review, we look at the effects of altered precipitation patterns – including reductions and increases in average rainfall, and changes in rainfall frequency – on grassland invertebrates and the plant communities they inhabit. We focus on findings from field-based/observational studies and precipitation manipulation experiments conducted in grasslands, including steppe and savannah habitats.

**Table 1:** A summary of the major precipitation manipulation experimental platforms assessing both plant and invertebrate responses to altered rainfall regimes.

Name; Location; Climate	Manipulation	Ecosystem; Plant groups	Invertebrate groups; Collection method	Method; Shelter design	Outcome	Reference
Silwood (UK) Temperate, cool	+/- water, summer drought and winter increase	Grassland; Forbs and grasses	Auchenorrhyncha, Araneae, Coleopt., Collembola, Dipt., Heteropt., Isopoda; Vacuum	Irrigation with rain water; Removeable roof	Under rainfall and nitrogen addition, plants did not respond. In the third year plant biomass declined in drought plots. Auchenorrhyncha and Araneae declined with plant biomass.	Lee et al., 2014

Wytham – TIGER IV 2c. (UK) Temperate, cool	+/- water, +/- root herbivores	Calcareous grassland; Forb	Lepidopt., Coleopt.; Manual	Manual with deionised water; Mobile shelters	Enhanced summer rainfall increased leaf miner abundance, but not when root herbivores were also present. Root herbivores reduced the parasitism rates of moths above ground (smaller pupal size). Plants under drought were overall less susceptible to leaf-miners regardless of root damage.	Staley et al., 2007
			
	+/- water, + winter heat	Calcareous grassland; Forbs, legumes and grasses	Auchenorrhyncha; Vacuum sampling		Added water increased plant cover and Auchenorrhyncha abundance; though drought reduced vegetation cover, the abundance of Auchenorrhyncha remained at ambient levels.	Masters et al., 1998

BCNWR^1^ (USA) Subtropics, Warm/moderate cool	+ water, natural drought	Mixed-grass prairie and oak savannah; Forbs and grasses	Orthopt.; Manual	Water application method not mentioned; No shelter, natural drought	Water stress reduced plant biomass but not nutrient content and species diversity. Drought reduced forb protein content and grasshopper abundance and diversity. There was increased abundance and species richness of certain grasshoppers in increased precipitation plots.	Lenhart et al., 2015

OCCAM^2^ (USA) Temperate, cool	+/- heat, +/- water, +/- CO_2_	Old field – fescue; Forbs, legumes and grasses	163 morphospecies; Sticky traps, vacuum sampling	Irrigation with rain water; Fixed roof	No strong trends in terms of water effects; there was greater peak plant biomass in wet compared to dry. Weak effects of soil moisture on invertebrate community composition; more parasitoids in the dry treatment – temperature more important.	Villalpando et al., 2009

Agroscope (Switzerland) Temperate, cool	- water, diversity increments	Calcareous pasture; Forbs, legumes and grasses	Annelida; Mustard extraction	Not mentioned; Temporary shelter: summer only	Measurements were taken 1 year after drought application. Drought significantly increased the biomass of earthworms in plots where subordinate plant species were present. Drought caused shifts in earthworm community in terms of individual species.	Mariotte et al., 2016

Berkeley (USA) Subtropical, cool	+ water, winter precipitation +, spring precipitation +	Grassland; Forbs, legumes and grasses	Coleopt., Hemipt., Hymenopt., Orthopt., Araneae; Manual, pitfall	Irrigation with spring water; No shelter	Spring water addition caused diminishing increases in winter forbs/legumes resulting in lower plant and invertebrate species richness at the end of 5 years.	Suttle et al., 2007

DRIGrass^3^ (Australia) Subtropical, warm/moderate cool	+/- water, altered frequency, summer drought	Pasture; Forbs, legumes and grasses	Coleopt., Hemipt., Hymenopt., Orthopt., Araneae; Vacuum sampling, sticky trap	Automatic irrigation with tap water; Fixed shelter	TBD	Power et al., unpublished


*Climate classifications follow the Global Agro-Ecological Zones set out by the Food and Agriculture Organization (gaez.fao.org). ^1^Balcones Canyonlands National Wildlife Refuge (Marble Falls, TX, USA). ^2^Oldfield Community Climate and Atmospheric Manipulation (Oak Ridge, TN, USA). ^3^Drought and Root herbivore Impacts on Grassland (Richmond, NSW, Australia).*

## Invertebrate Responses to Precipitation Change

### Direct Impacts

In general, terrestrial arthropods are sensitive to changes in moisture, given their high surface-to-volume-ratio (Kimura et al., 1985). Under reduced rainfall, most aboveground arthropods avoid desiccation behaviorally by migrating, hiding in the soil, or, in a few cases, building a shelter (Willmer, 1982; Zalucki et al., 2002; Berridge, 2012). Structurally speaking, soft-bodied arthropods (isopods and myriapods) lack the waxy cuticle found in arachnids and insects that prevents or reduces evaporation (Berridge, 2012). This, in combination with differences in excretion-related water losses (Horne, 1968), suggests that soft-bodied arthropods will be more vulnerable to reductions in water availability, and, in some cases, to increases (Sylvain et al., 2014). Thus, changes in rainfall could be expected to affect hard and soft-bodied groups differently, resulting in shifts in the arthropod community.

On the other end of the spectrum, average increases in rainfall may negatively impact arthropods by disrupting flight, reducing foraging efficiency and increasing migration times (Peng et al., 1992; Drake, 1994; Kasper et al., 2008). Some arthropods can vary their behavior to combat the effects of extreme rainfall events and flooding by shelter-seeking and utilizing submersion tolerance strategies (Lambeets et al., 2008). The effects of increased rainfall on arthropods are also dependent on invertebrate morphology and are group-specific, with larger winged insects like Lepidopterans having a much higher degree of ‘unwettability’ (i.e., requiring greater volumes of water to become wet) than smaller winged insects (Wagner et al., 1996). Altered rainfall frequency can be positive or negative for invertebrates depending on the size of the event (Nielsen and Ball, 2015), but on the whole is expected to impact more rain-sensitive orders like Lepidoptera (Palmer, 2010).

Arthropods that spend all or some of their life stages belowground have evolved behaviors to manage water stress in times of drought and flood (Verhoef and Witteveen, 1980). Under reduced water availability, most soil invertebrates combat fluctuating moisture by relocating to places that are more favorable within the soil-matrix. Such movement, however, is dependent on suitable soil moisture and texture (Lees, 1943; Brust and House, 1990). Some invertebrates build earthen chambers, controlling the microclimate, similar to shelter-builders aboveground (Haile, 2001; Barnett and Johnson, 2013). Prolonged drought conditions may favor those species capable of such behaviors. Similarly, larvae with morphological adaptations to flooding may fare better in areas predicted to experience increases in rainfall. Species that have evolved in flood-prone environments in particular, like the cranberry root grub, with water-repellent hairs along its body that can trap air (King et al., 1990; Villani et al., 1999), may stand to have competitive advantages over flood-intolerant species. Thus, invertebrates both above and belowground have evolved a range of behavioral and morphological adaptations to alterations in water availability. Differences in the use of such strategies between species and functional groups will likely lead to alterations in invertebrate community composition.

### Indirect (Plant-Mediated) Impacts

#### Invertebrate Responses to Plant Quantity and Quality

There is strong evidence in the literature for resource quantity-driven changes in invertebrate herbivore populations under altered precipitation regimes. Reduced rainfall results in reduced plant biomass, aboveground net primary productivity (ANPP), forage quality and cover, with increases in canopy light penetration and root:shoot ratios (Fay et al., 2003; Wu et al., 2011), leaving less plant biomass to support herbivores; however there is strong evidence that this response is ecosystem dependent (Byrne et al., 2013). Accordingly, declines have been reported – across various ecotypes – in the abundances of Orthoptera (Kemp and Cigliano, 1994); earthworms and scarabs (Davis et al., 2006; Mariotte et al., 2016); belowground herbivores (Staley et al., 2007); and across herbivore communities generally (Lee et al., 2014). Plant quality changes could also play a role in these declines. In a feeding experiment, army worm larvae reared on droughted Yorkshire fog grass took longer to develop and had higher mortality rates than those feeding on non-droughted grass, due to lower soluble protein content (Walter et al., 2012). While some trends can be identified in the responses of invertebrates to reductions in rainfall, there is a high degree of variation between species. For instance, gastropod species in a UK study had highly individual responses to changes in water availability, with some benefitting from drought and others instead occurring in greater abundance under supplemented rainfall (Sternberg, 2000).

Increases in average precipitation (to a degree – the negative effects of flooding in grasslands have been reviewed elsewhere – see Plum (2005)) may result in benefits to invertebrate herbivores, except in cases where increased moisture facilitates pathogens and disease (Grant and Villani, 2003). On the whole, increases in precipitation lead to increases in ANPP (Zaller and Arnone, 1999; Byrne et al., 2013). Consequently, studies have reported improved grasshopper nymph survival (Guo et al., 2009) and increased abundance and richness of grasshoppers (Lenhart et al., 2015). However, in contrast to the increases in grasshopper abundance reported in Lenhart et al. (2015) two other studies reported reductions in grasshopper survival under similar increased rainfall treatments (Barton et al., 2009; Guo et al., 2009).

Hence, a recurrent theme in the literature is that the responses of herbivorous invertebrates to altered precipitation will likely be idiosyncratic in nature, making it difficult to make generalized predictions about the directions of their responses under different scenarios (González-Megías and Menéndez, 2012; Nielsen and Ball, 2015). The responses of herbivores to both reduced and increased water availability are likely to be linked to the responses of their individual food-plant(s), as well as the invertebrate specie’s own physiological precipitation optimum (Schowalter et al., 1999).

#### Invertebrate Responses to Plant Community Composition

The responses of a grassland plant community to alterations in rainfall depend on the type of grassland (i.e., average water state – mesic, xeric etc.; Heisler-White et al., 2009), as well as the plant functional types (PFTs) that dominate the system (Collins et al., 2012; Andrey et al., 2014). For example, under altered rainfall frequency, with longer dry periods between more intense rainfall events, mesic grasslands generally experience a decrease in ANPP, whereas xeric grasslands show an increase (Fay et al., 2002, 2003; Heisler-White et al., 2009). In terms of PFTs, grasslands dominated by C_4_ grasses tend not to show stimulations in ANPP under increased rainfall, perhaps because they are likely to be less water limited than their C_3_ counterparts (Niu et al., 2008; Wu et al., 2011; Wilcox et al., 2015). Indeed, there is evidence that herbivorous insects consume relatively more C_4_ plants in years with reduced rainfall frequencies (Warne et al., 2010), possibly due to improved quality or increased quantities of these plants under such scenarios. Thus, we could expect that reorganizations occurring at the plant community level in response to altered rainfall regimes will have consequences for herbivores, particularly for specialist feeders which may be reliant on the presence of just one or two plant species.

So far, experimental evidence directly linking precipitation-mediated changes in plant diversity to changes in the herbivore community is lacking. However, a 5-year field experiment by Suttle et al. (2007) showed that whilst increased summer rainfall enhanced plant biomass, increased dominance and reduced grassland plant species richness had eventual negative consequences for the invertebrate community. Specifically, herbivore and consumer abundance declined and the invertebrate food web became simplified, potentially pointing to the loss of more specialized herbivores. This study demonstrates the importance of longer-term studies in detecting plant community shifts as opposed to more immediate biomass responses. Furthermore, Wilcox et al. (2016) recently showed that short-term plant community shifts in response to increased water availability may be misleading when considering shifts over a decadal time scale.

#### Secondary Consumer Responses to Altered Rainfall

Alterations occurring in the abundance and diversity of primary consumers can flow up through the food chain to affect populations of predators and parasitoids (Suttle et al., 2007; Lee et al., 2014), which may themselves be more sensitive to climatic change (Voigt et al., 2003). Buchholz et al. (2013) found reductions in semi-dry grassland spider and carabid diversity and abundance under water-limited conditions. However, at a similar site 3 years earlier, the same authors found no change in spider species richness, composition or abundance under precipitation manipulation (Buchholz et al., 2010). Similarly, in a Chinese steppe, reduced rainfall caused declines in herbivore abundance with no corresponding decline in secondary consumers (Zhu et al., 2014). Clearly, as with herbivores, there will be differences in the individual responses of higher trophic levels to changes in precipitation patterns.

#### Precipitation-Sensitive Species Interactions

The idiosyncratic nature of invertebrate responses can be at least partially explained by complex precipitation-driven alterations in the interactions occurring between species within the system. The handful of studies which have tackled pairwise species interactions under precipitation manipulations have found complex, unpredictable responses with the potential to affect multiple trophic levels. For instance, spatially separated above- and belowground herbivores may influence each other through their effects on the shared host plant, such as by altering secondary chemistry (Johnson et al., 2012). Staley et al. (2007) found that enhanced summer rainfall increased the abundance of leaf mining moths on wild basil, but not when root herbivores were present. The negative effects of root herbivores on leaf miner pupal size reduced the parasitism rates of moths above ground, indicating the potential for precipitation-altered species interactions to have knock-on consequences for higher trophic levels.

In a separate study, detritivorous tenebrionid beetles belowground had negative effects on the abundances of generalist sap sucking and chewing herbivores when summer precipitation was supplemented, similar to the findings of Staley et al. (2007) (González-Megías and Menéndez, 2012). In contrast, the presence of belowground herbivores had positive impacts on aboveground leaf-mining flies, restoring their pupal weight and development time to ambient levels, when reared under drought conditions on milk-thistle (Staley et al., 2008). Taken together, these studies suggest that belowground organisms could serve to moderate the effects of reduced or increased water availability on aboveground herbivores, which may otherwise be expected to decrease or increase in abundance, respectively, in response to such rainfall regimes. As with the responses of individual species, the directions of the responses of the interactions between multiple species may also prove to be species- and system-specific. Further work is needed in the area to determine whether or not generalizations can be made, and to determine whether other interactions such as competition may also be affected by alterations in precipitation.

## Invertebrate-Mediated Feedbacks on Plant Communities

At the ecosystem scale, invertebrate herbivores generally exert weak control over grassland plant communities (Whiles and Charlton, 2006; Coupe et al., 2009), though their impacts on plant species richness, for instance, may be stronger during herbivore outbreaks (Olff and Ritchie, 1998). Altered precipitation has the capacity to change the relative strength of the interactions occurring between grassland plant and invertebrate communities, by altering the abundance and composition of species within the system. In a Canadian grassland, invertebrates caused short-term reductions in plant cover, increases in root mortality and altered plant composition compared with pesticide treated plots (Coupe et al., 2009). The effects of the invertebrate community only became apparent under naturally occurring drought conditions. This suggests that invertebrate herbivores may exacerbate the negative effects of drought for grassland plants, and that grasslands may become more vulnerable to herbivores under drought.

In an American temperate old-field study, experimentally increased rainfall caused declines in grasshopper abundance, which translated into a 15% reduction in grasshopper-inflicted plant damage for every 1 cm of increase in mean monthly precipitation (Barton et al., 2009). Conversely, in a limestone grassland, the plant community sustained an increase in biomass under supplemented rainfall scenarios, despite a significant increase in the abundance of Auchenorrhyncha herbivores (leaf, plant, and frog hoppers; Masters et al., 1998). Assuming that this greater abundance of insects inflicted comparable levels of damage on a per capita basis as those in ambient plots, this would suggest that grassland plants may be able to maintain increased growth despite higher levels of herbivory under increased rainfall scenarios. These two studies demonstrate that the strength of indirect, invertebrate community-mediated effects of altered precipitation on grasslands will depend on the identities of both the plant community and invertebrate species involved.

## Conclusion and Future Directions

**FIGURE 1:**
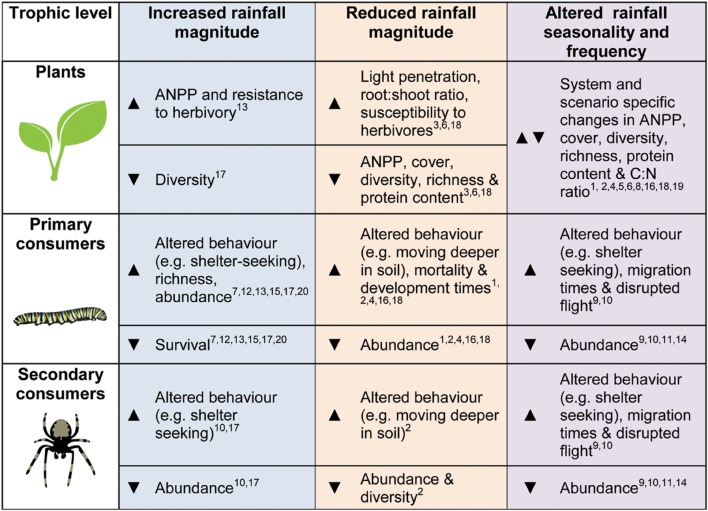
**A summary diagram of the general trends expected or found in the literature (theoretical, experimental, and observational studies) for plant, primary consumers (herbivores, detritivores etc.) and secondary consumers (predators and parasitoids), in response to altered precipitation regimes.** Arrows: 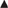 indicates increases in the given metric, 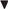 represents declines and 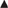 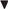 denotes more varied results. References are given by the numbers on the diagram: (1) Barnett and Johnson (2013), (2) Buchholz et al. (2013), (3) Coupe et al. (2009), (4) Davis et al. (2006), (5) Fay et al. (2002), (6) Fay et al. (2003), (7) Guo et al. (2009), (8) Heisler-White et al. (2009), (9) Kasper et al. (2008), (10) Lambeets et al. (2008), (11) Lee et al. (2014), (12) Lenhart et al. (2015), (13) Masters et al. (1998), (14) Palmer (2010), (15) Staley et al. (2007), (16) Staley et al. (2008), (17) Suttle et al. (2007), (18) Walter et al. (2012), (19) Warne et al. (2010), (20) Zhu et al. (2014).

Depending on the underlying water-status of the ecosystem, alterations in rainfall may have generally negative direct and indirect consequences for invertebrates (summarized in **Figure 1**). Changes in precipitation will also have the potential to cause impacts spanning multiple trophic levels, moderating the outcomes of species interactions. Reductions in rainfall may exacerbate the negative effects of herbivores for the plant communities they inhabit, though other players in the system might alter this response (e.g., Staley et al., 2008). In order to better understand how grassland invertebrates – and the important ecological processes they underpin – will respond to altered precipitation, we highlight the following four areas for future research:

(1) The incorporation of invertebrate responses in the design of current and future precipitation manipulation experiments: invertebrate responses remain under-studied in rainfall manipulation experiments, with the majority of studies considering only the responses of plants to short-term rainfall alterations of limited scope – altered frequency scenarios, for instance, remain critically under-represented (Johnson et al., 2016). This under-representation, coupled with the idiosyncratic nature of the responses detailed to date, makes it difficult to identify solid trends and predict how grasslands will respond to a wide range of precipitation scenarios. Achieving such a goal will require an increased number of studies from which to draw patterns from, across a broader range of precipitation scenarios.
(2) A focus on long-term studies: aside from the identities of the different components of the system, the timescale over which precipitation alterations are studied may also be important. The relatively short term nature of many field experiments to date obfuscates our ability to make more realistic predictions about how grassland communities will respond to future changes (Beier et al., 2012). Such studies are needed in order to capture changes in the direction of responses over time and lags in the manifestation of responses – particularly given the potential for short-term studies to have misleading results compared with those over longer timescales (Suttle et al., 2007; Wilcox et al., 2016).
(3) Greater geographical representation: this should be prioritized to determine the extent to which findings can be extrapolated across different biomes (the studies we review here are mostly from the UK and USA; Beier et al., 2012; White et al., 2012). Given how many plant and insect responses are likely to depend on the underlying water-status of the system, research across ecotypes will be an essential target for enabling progress the field.
(4) Examination of multiple climate factors at once: there is a need for experiments reflecting the reality of global change which will involve the simultaneous alterations of many factors (Villalpando et al., 2009; Beier et al., 2012). This is especially important given the potential for synergisms between factors, as may be expected between increased temperatures and reduced water availability. On the other hand, the effects of one factor may serve to moderate those of another (e.g., Lee et al., 2014).

Such studies will not be without logistical difficulty, though future developments in technology will help to ease this, including improvements in long-term, sensor-based data gathering. Continued development of DNA-based methods like metabarcoding will assist community-level studies by reducing time-consuming work and taxonomic expertise (Cristescu, 2014). Results from studies like those suggested above will provide critical information about grassland community responses for use in theoretical modeling approaches such as structural equation modeling, enabling the testing of theories at scales not yet possible experimentally. Such experimental and modeling approaches, carried out with broader geographical and precipitation-scenario representation, will be necessary in order for us to form more accurate predictions about the fate of ecologically important grassland ecosystems under climate change.

## Author Contributions

KB and SF contributed equally in drafting, writing, and approving the final manuscript.

## Conflict of Interest Statement

The authors declare that the research was conducted in the absence of any commercial or financial relationships that could be construed as a potential conflict of interest.
